# A Novel Method for Evaluating the Cardiotoxicity of Traditional Chinese Medicine Compatibility by Using Support Vector Machine Model Combined with Metabonomics

**DOI:** 10.1155/2016/6012761

**Published:** 2016-08-23

**Authors:** Yubo Li, Haonan Zhou, Jiabin Xie, Mayassa Salum Ally, Zhiguo Hou, Yanyan Xu, Yanjun Zhang

**Affiliations:** ^1^Tianjin State Key Laboratory of Modern Chinese Medicine, School of Traditional Chinese Materia Medica, Tianjin University of Traditional Chinese Medicine, 312 Anshan West Road, Tianjin 300193, China; ^2^Tianjin State Key Laboratory of Modern Chinese Medicine, Tianjin University of Traditional Chinese Medicine, 312 Anshan West Road, Tianjin 300193, China

## Abstract

Traditional biochemical and histopathological tests have been used to evaluate the safety of traditional Chinese medicine (TCM) compatibility for a long time. But these methods lack high sensitivity and specificity. In the previous study, we have found ten biomarkers related to cardiotoxicity and established a support vector machine (SVM) prediction model. Results showed a good sensitivity and specificity. Therefore, in this study, we used SVM model combined with metabonomics UPLC/Q-TOF-MS technology to build a rapid and sensitivity and specificity method to predict the cardiotoxicity of TCM compatibility. This study firstly applied SVM model to the prediction of cardiotoxicity in TCM compatibility containing* Aconiti Lateralis Radix Praeparata* and further identified whether the cardiotoxicity increased after* Aconiti Lateralis Radix Praeparata* combined with other TCM. This study provides a new idea for studying the evaluation of the cardiotoxicity caused by compatibility of TCM.

## 1. Introduction

In the early research, Li et al. have found 10 highly specific early cardiac toxicity biomarkers and established a support vector machine (SVM) prediction model [1]. And they used the SVM model to predict the cardiotoxicity of drugs and traditional Chinese medicine (TCM); results showed that the SVM prediction model had a better sensitivity and specificity. However, currently there is no effective method for evaluating the cardiotoxicity of TCM compatibility. And it is still not known how the SVM model can be applied to predict cardiotoxicity of TCM compatibility. So we hope to develop a new approach with high specificity and accuracy for prediction of TCM compatibility.

For thousands of years, ancient Chinese people gradually formed the medical theory system of traditional Chinese medicine (TCM), in the process of struggling with the diseases [2]. Compatibility of TCM is the main form of clinical treatment; we always used two or more TCM to produce synergies effect or improve therapeutic effect [3].* Aconiti Lateralis Radix Praeparata* (fuzi in Chinese) is one of the commonly traditional Chinese medicines used in clinical treatment; modern research has shown that fuzi has lots of efficacy, such as cardiotonic, anti-inflammatory, analgesic, antimyocardial ischemia, and hypoxia effect. The alkaloid components of fuzi may cause toxic reactions, especially the cardiotoxicity, so it has been always used in the combination with other Chinese herbs to reduce its toxicity in clinical treatment [4, 5]. Research has shown that fuzi combined with* Zingiberis rhizoma* (ganjiang in Chinese) can enhance the effect of the treatment of acute heart failure [6]. Other studies have found that, for fuzi and* Rhei Radix Et Rhizoma* (dahuang in Chinese), these kinds of combinations can reduce the toxicity of fuzi and produce other synergistic effects at the same time [7]. However, if TCM compatibility is improper, it will produce toxic effects and even cause damage to the human body; we called this phenomenon “incompatibility” [8].

With the development of times and people growing awareness of drug safety, incompatibility of TCM increasingly received widespread attention. In recent years, medical workers conducted series of studies on the toxicity of TCM incompatibility; it aimed to provide the basis for clinical application. Currently, researchers commonly used biochemical and histopathological tests to evaluate the safety of TCM incompatibility in clinical treatment [9]. But due to the complex material composition of TCM, variety of metabolic pathways, and different targets, traditional evaluation methods have some limitations. Many biological indicators are lack of high sensitivity and specificity, small and weak elaboration of toxicity mechanisms, and slowness of research. In addition, traditional methods require significant time, energy, and laboratory animals [10, 11]. Thus, we need a detection method with less damage, strong specificity, and high accuracy to study the incompatibility of TCM.

Metabonomics is a new research method with comprehensive analysis. We can explore variation and characteristics of drug metabolism in the body by analyzing metabolites information in various biological samples and further on to evaluate drug efficacy, predict drug toxicity, and diagnose disease [12]. Metabonomics technology also can monitor the changes process of drug metabolism in the body, observe the changes of endogenous metabolites at the same time, and then infer the mechanism of TCM. Moreover, we can look for or clarify the drugs target or receptor of toxic effects through the terminal information of metabolites. However, metabonomics technologies produce large and multidimensional data with diversity formats; this leads to a heavy workload and time-consuming data preprocessing [13]. Support vector machine (SVM) as a method with good generalization ability has obvious advantages in application of metabonomics data processing. Because the final decision function of SVM only determined by few support vectors and few support vectors determines the final result, so it helps us seize the key sample and exclude a large number of redundant sample [14, 15]. This method is simple and convenient and it can make the research results more reliable. Thus, we used SVM to analyze experimental data combined with metabonomics.

In this study, based on the preestablished cardiac toxicity SVM model, we apply it to predict cardiotoxicity of TCM compatibility containing fuzi; then we combine biochemical and pathology tests to evaluate the cardiotoxicity and analyze whether the cardiotoxicity increased or not. The novel method is effective in rapidly and precisely predicting the cardiotoxicity of TCM compatibility. In addition, this study also provides a new way for the evaluation of toxicity in TCM compatibility.

## 2. Materials and Methods

### 2.1. Extraction of Traditional Chinese Medicine

In this study, single herb groups, processed* Aconiti Lateralis Radix Praeparata* (heishunpian in Chinese, HSP),* Trichosanthis Fructus* (gualou in Chinese, GL),* Fritillariae Thunbergii Bulbus* (zhebeimu in Chinese, ZBM),* Ampelopsis Radix* (bailian in Chinese, BL), and* Bletillae Rhizoma* (baiji in Chinese, BJ), respectively, take 200 g. Compatibility groups take 200 g heishunpian, respectively, combined with the same amount of gualou, zhebeimu, bailian, and baiji. All of these were extracted twice with 10 and 8 times the amount of pure water under reflux for 60 minutes, respectively. Then the extracting solutions were filtrated, combined, and concentrated to 1 g/mL (amount of crude drug) aqueous extract samples.

### 2.2. Animal Treatment

The experimental animals were supplied by the Academy of Military Medical Sciences Experimental Animal Centre (Beijing). 110 male Wistar rats, with body weight of 200 ± 20 g, were housed in Tianjin Institute of Radiation Laboratory Animal Center of SPF Animal Laboratory for one week. All of the rats were randomly divided into 10 groups; the control group has twenty rats and each of the model groups has ten rats. The growth environment was as follows: 12-hour day and night turnover, ambient temperature of 23 ± 2°C, and humidity of 35 ± 5%.

To reduce the pain of animals, all experiments were carried out in accordance with Chinese national laws and local guidelines. The animal study was approved by the Animal Ethics Committee of Tianjin University of Traditional Chinese Medicine under approval number TCM-2012-078F01.

### 2.3. Sample Collection and Preparation

All animals were only given water without food for 12 h before sample collection. After each group was intragastrically given corresponding water extract of single herbal medicine (10 g/kg), water extract of compatible TCM (10 g/kg), and the normal saline (10 mL/kg) for seven days [16, 17], we took 10 mL of abdominal aorta blood and heart tissue in rats. 5 mL of whole blood was centrifuged at 3500 rpm for 8 minutes. The obtained supernatant is plasma, and it was stored in −80°C refrigerator for metabonomics research. Another 5 mL of whole blood was placed in normal tubes and then handled and stored in the same conditions for biochemical parameters test. The heart tissue was immersed in 10% formaldehyde solution for pathological examination by haematoxylin and eosin (H&E) staining.

For H&E staining, the heart tissues were trimmed and embedded in paraffin wax. Then, 5 *μ*m thick slices were cut and affixed to glass slides. The slices were deparaffinized with xylene, hydrated, stained with haematoxylin for 10 mins, differentiated with hydrochloric alcohol, stained with eosin, and dehydrated in a graded alcohol series and then cleaned with xylene. Finally, the histopathological changes were observed by a light microscopy at 100x and 200x magnification.

### 2.4. Chromatographic and Mass Spectrometric Conditions

In this study, ACQUITY UPLC HSS C18 column (2.1 × 100 mm, 1.7 *μ*m, Waters) was used for analysis of plasma samples. The volume of plasma injection is 5 *μ*L, the column temperature was 40°C, and the flow rate was 0.3 mL/min. Mobile phase A (0.1% formic acid in water) and mobile phase B (0.1% formic acid in acetonitrile) were used for gradient elution, and the specific conditions were as follows: 0–0.5 min, 99% A; 0.5–2 min, 99% A–50% A; 2–9 min, 50% A–1% A; 9-10 min, 1% A; 10–10.5 min, 1% A–99% A; 10.5–12 min, 99% A. Mass spectrometry analysis was conducted by electrospray ionisation in positive mode. High-purity N_2_ was used as auxiliary ionization and desolvation gas, and the MS parameters were as follows: drying gas flow rate was set to 10 mL/min, temperature of N_2_ was set to 325°C, pressure of atomized gas was 350 psi, flow rate of desolvation gas was 600 L/h, capillary voltage was 3.5 kv, and quadrupole scan range was *m*/*z* 50–1000. All samples were randomly injected.

In addition, we singled out the plasma samples from each group and mixed them together to make quality control (QC) samples. Then we injected blank and QC samples every 10 samples to test system precision, method precision, and sample stability.

### 2.5. Data Process

Independent sample* t*-test was used to calculate the difference between the control group and the models group. The process of the calculation is as follows. First, we ensured that the two populations obeyed the normal distribution, and the samples were independent mutually. Then the* t*-test was used to test whether there is a significant difference between the two populations. All the calculation was completed by SPSS software.

The retention time and the relative content of the metabolites differed within the spectrum. Twenty of them were randomly selected to evaluate the relative standard deviations (RSD) of precision and reproducibility. The raw data of the control and model groups were collected and exported by MarkerLynx Version 4.1 (Waters Corp., Manchester, USA). Then according to the retention time, *m*/*z* values, and corresponding MS^2^ information [1], we found out ten predetermined cardiac toxicity biomarkers in the exported data. The variation trends of cardiac toxicity biomarkers were investigated. Finally, we used the preestablished SVM model to predict the cardiac toxicity in both single herb groups and compatibility groups.

## 3. Results and Discussion

### 3.1. Biochemical Analysis and Histopathological Assessment

**Figure 1:**
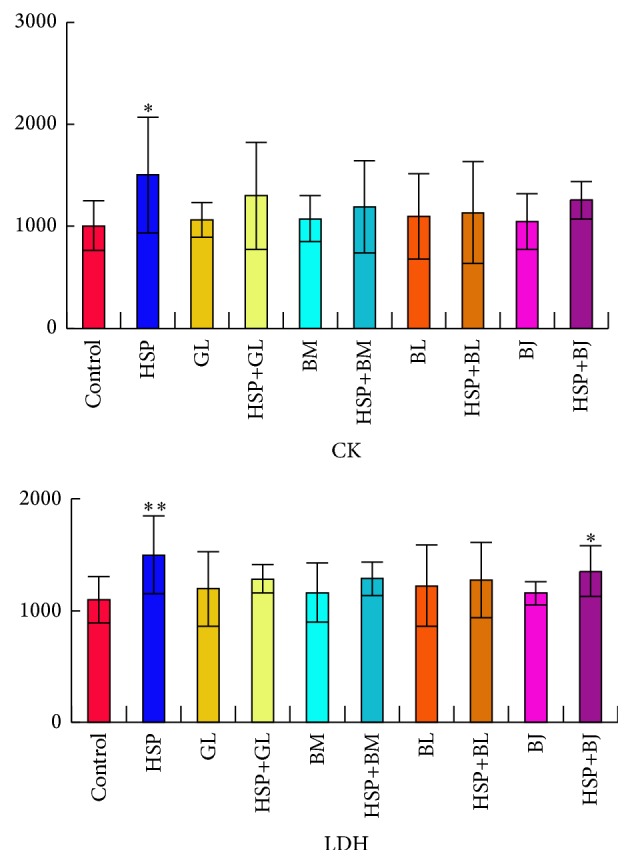
Content changes of biochemical indices (CK, LDH) in rats, respectively, comparing model groups with NS group (^*∗*^ *p* < 0.05, ^*∗∗*^ *p* < 0.01).

In this study, creatine kinase (CK) and lactate dehydrogenase (LDH) were selected as biochemical indices to evaluate whether the drug caused damage to the heart [18–22]. Biochemical results are shown in Figure 1. From the result, we can see that CK and LDH were significantly increased in heishunpian group compared with the control group. It means heishunpian shows significant cardiac toxicity. And there was no significant difference of CK and LDH in other single herb groups; it means zhebeimu, gualou, bailian, and baiji did not show the cardiac toxicity. Furthermore, compared with single herb groups, CK and LDH were increased in various degrees in compatibility groups. But they were not higher than heishunpian group when compared with it.

Compared with the control group, LDH was significantly increased in heishunpian-baiji group, but CK did not show significant difference. It is not enough to suggest that there was cardiac toxicity after combination. In addition, CK and LDH did not show significant difference in other compatibility groups. This indicated that there was no cardiac toxicity after heishunpian, respectively, combined with zhebeimu, gualou, bailian, and baiji.

**Figure 2:**
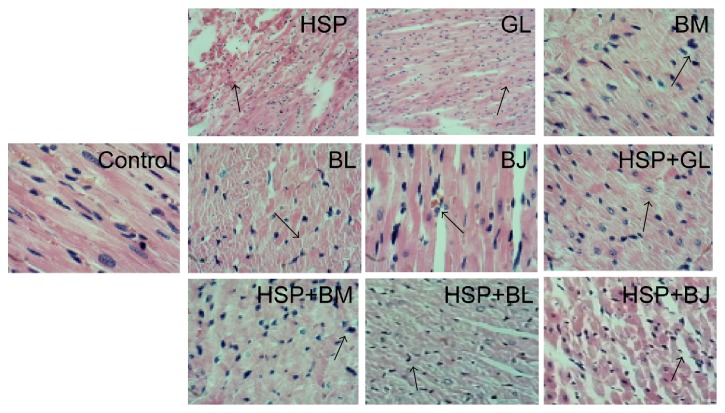
Histopathological results of the heart by H&E staining. Control group: no necrotic lesions; HSP: heishunpian group, infiltration of lymphocytes; GL: gualou group, irregular arrangement of cell nuclear; BM: beimu group, inhomogeneous staining of cytoplasm; BL: bailian group, cytoplasm discoloration and uneven staining; BJ: baiji group, overflow of red blood cells in muscle fibers; HSP + GL: heishunpian-gualou group, uneven staining of cytoplasm; HSP + BM: heishunpian-beimu group, mild hyperchromatic of cell nuclear; HSP + BL: heishunpian-bailian group, loss of muscle fibers and irregular arrangement of cell nuclear; HSP + BJ: heishunpian-baiji group, atrophy of striated muscle.

We used histopathological examination to evaluate the extent of heart damage. The histopathological results were shown in Figure 2. Compared with the NS group, all the single herb groups and compatibility groups exhibited normal basic structure of rat heart tissue and arranged regular cardiac muscle fibers. In heishunpian group, muscle fibers were seen scattered with small lymphocytes, myocardial stripes existing, and part of the heart cytoplasm staining shades. In other single herb groups, the arrangement of cell nuclear was irregular, and endochylema staining was uneven. In the compatibility groups, a small amount of the dilated blood vessels could be seen under myocardial tunica, striated muscle fibers were visible, myocardial cytoplasmic staining was slightly uneven, and cell nuclear showed a mild shift with different sizes.

### 3.2. Biomarkers for the Early Prediction of Cardiotoxicity

The RSD of peak areas and retention times of 20 randomly selected chromatographic peaks were less than 15%, which indicates that the sample detection method meets metabolomics requirements. And the QC results of this study were shown in Table 1. At present, myocardial enzymes were commonly used as indicators of heart disease. But these indicators always show significant changes when heart tissue pathological damage occurred. This method showed a lag nature and lack of sensitivity and specificity [23, 24]. Based on the early study, combined with retention time, *m*/*z* values, and corresponding MS^2^ information, we found out ten early cardiac toxicity biomarkers from metabolomic data; the information of those biomarkers was shown in Table 2. Then compared with NS group, we analyzed the variation trends of ten early cardiac toxicity biomarkers in model groups. Results showed that the content of L-carnitine in heishunpian, zhebeimu, bailian, and compatibility groups was higher than NS group and the content of other biomarkers in those groups was lower than NS group. The variation trends of these biomarkers were consistent with the results of early experiment [1]; detailed information is shown in Table 3.

### 3.3. Prediction Results of SVM Model Analysis

**Figure 3:**
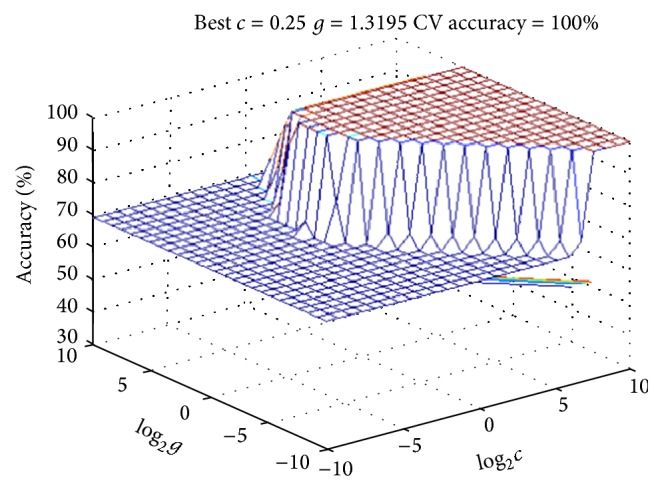
Three-dimensional view of the SVM model of ten biomarkers: comparing single herb groups with NS group (the parameters are described in the following: Best *c* = 0.25, Best *g* = 1.3195, and CV accuracy = 100%).

**Figure 4:**
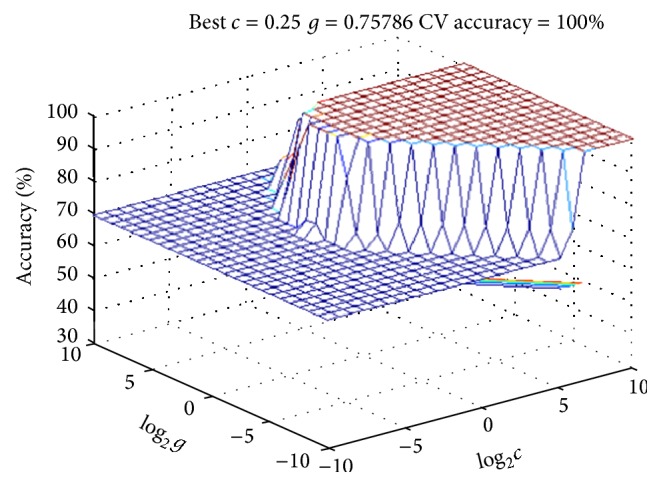
Three-dimensional view of the SVM model of ten biomarkers: comparing compatibility groups with HSP group (the parameters are described in the following: Best *c* = 0.25, Best *g* = 0.75786, and CV accuracy = 100%).

The obtained metabonomics data were analyzed by using the preestablished cardiac toxicity SVM model. First, the data of control group were used as training set, and the data of single herb groups were used as test set; then the SVM model was run to predict if there is cardiac toxicity in single herb groups. Next, we replaced the control group with heishunpian group; that is, the data of heishunpian group were used as training set, and the data of compatibility groups were used as test set. Then we ran SVM model to predict if there is cardiac toxicity in compatibility groups. 3D views and parameters of SVM model were shown in Figures 3 and 4.

SVM prediction results were described in Table 4. From the results, it can be seen that all the single herb groups did not show cardiac toxicity except heishunpian group. It indicated that the bodies were exposed to cardiac toxicity in heishunpian group, and this was consistent with biochemical results. In addition, all the compatibility groups showed cardiac toxicity when heishunpian group was used as control group; it means that there is cardiac toxicity, and the cardiac toxicity was increased after heishunpian combined with zhebeimu, gualou, bailian, and baiji. This was consistent with early reports. Huang et al. have reported that when heishunpian combined with gualou, zhebeimu, bailian, and baiji, the content of diester alkaloids was obviously increased [25]. Ma et al. have found that the codecoctions of fuzi combined with gualou increased the cardiotoxicity [26]. Moreover, Bian et al. reported that the the content of toxic components was increased after fuzi combined with zhebeimu [27].

## 4. Conclusions

In this study, we used UPLC-Q-TOF-MS metabolomics analysis, combined with preestablished SVM model to predict the cardiac toxicity of heishunpian, gualou, zhebeimu, bailian, and baiji and determine whether the cardiac toxicity increased after heishunpian combined with gualou, zhebeimu, bailian, and baiji. According to biochemical and pathological results, we found that heishunpian group showed obvious cardiac toxicity, and compatibility groups did not show significant cardiac toxicity. But SVM model prediction results revealed that the cardiac toxicity were increased in all compatibility groups compared with heishunpian group. It indicates that traditional testing methods have not yet showed cardiac toxicity. This may be due to lack of time, so the cardiac toxicity was not exposed enough. Therefore, compared with traditional detection methods, early cardiac toxicity biomarkers, and the preestablished cardiac toxicity SVM model can rapidly and accurately predict toxicity when heart tissue damage has not yet appeared. The discovery of early cardiac toxicity biomarkers has great significance for early predicting toxicity. Moreover, SVM model based on early cardiac toxicity biomarkers offers a new research direction for the prediction of cardiac toxicity in TCM compatibility. This study makes a theoretical support for cardiac toxicity studies and the application of cardiac toxicity biomarkers. It also provides a more reliable guidance for clinical application in early diagnosis of cardiac toxicity.

## Figures and Tables

**Table 1:** The results of experimental methodology.

Experiment name	RSD (retention time)	RSD (peak area)
Precision instrument	<0.48%	<11.0%
Method repeatability	<0.36%	<4.0%
Sample stability	<1.2%	<9.6%

**Table 2:** Ion information of early cardiotoxicity biomarkers [1].

*T*_*R*_ (min)	Obsd *m*/*z*	Calcd *m*/*z*	Error (ppm)	Metabolite	Formula	MS/MS
0.76	162.1123	162.1130	4.32	L-carnitine	C_7_H_15_NO_3_	162.1, 103.0
4.19	190.0863					
4.19	172.0755					
4.19	130.065					
4.67	347.2214	347.2222	2.30	19-Hydroxydeoxycorticosterone	C_21_H_30_O_4_	385.3, 369.2, 347.2, 329.2, 109.1, 97.1
5.45	432.3107					
5.87	299.2002					
7.30	468.3467					
7.85	548.3715	548.3716	0.18	LPC (20:2)	C_28_H_54_NO_7_P	570.3, 548.3, 184.1, 104.1
6.48	490.2913	490.2910	−0.61	LPC (14:0)	C_22_H_46_NO_7_PNa	490.2, 468.3, 184.1, 104.1

**Table 3:** Content change trend of early cardiac toxicity biomarkers.

*T*_*R*_ (min)	*m*/*z*	Metabolite	Content change									
			Identified	HSP	GL	ZBM	BL	BJ	HSP + GL	HSP + ZBM	HSP + BL	HSP + BJ
0.76	162.1123	L-carnitine	↑	↑	↓	↑	↑	↓	↑	↑	↑	↑
4.19	190.0863		↓	↓	↓	↓	↓	↓	↓	↓	↓	↓
4.19	172.0755		↓	↓	↓	↓	↓	↓	↓	↓	↓	↓
4.19	130.065		↓	↓	↑	↓	↓	↓	↓	↓	↓	↓
4.67	347.2214	19-Hydroxydeoxycorticosterone	↓	↓	↑	↓	↓	↓	↓	↓	↓	↓
5.45	432.3107		↓	↓	↓	↓	↓	↓	↓	↓	↓	↓
5.87	299.2002		↓	↓	↓	↓	↓	↓	↓	↓	↓	↓
7.30	468.3467		↓	↓	↓	↓	↓	↓	↓	↓	↓	↓
7.85	548.3715	LPC (20:2)	↓	↓	↓	↓	↓	↓	↓	↓	↓	↓
6.48	490.2913	LPC (14:0)	↓	↓	↓	↓	↓	↓	↓	↓	↓	↓

**Table 4:** SVM cardiac toxicity prediction results.

Single herb group	Prediction result	Compatibility group	Prediction result
HSP	1	—	—
GL	0	HSP + GL	1
ZBM	0	HSP + ZBM	1
BL	0	HSP + BL	1
BJ	0	HSP + BJ	1

“0” indicated that the TCM did not induce the cardiac toxicity; “1” indicated that the TCM induced cardiac toxicity.

## References

[1] Li Y. B., Ju L., Hou Z. G. (2015). Screening, verification, and optimization of biomarkers for early prediction of cardiotoxicity based on metabolomics. *Journal of Proteome Research*.

[2] Liu B. Y., Zhang Y. H., Hu J. Q., He L. Y., Zhou X. Z. (2011). Thinking and practice of accelerating transformation of traditional Chinese medicine from experience medicine to evidence-based medicine. *Frontiers of Medicine*.

[3] Zhang J.-H., Zhu Y., Fan X.-H., Zhang B.-L. (2015). Efficacy-oriented compatibility for component-based Chinese medicine. *Acta Pharmacologica Sinica*.

[4] Li Y. B., Hou Z. G., Wang Y. M. (2015). Screening and verification of linearly dependent biomarkers with acute toxicity induced by Aconiti Radix based on liquid chromatography-mass spectrometry-based metabolite profiling. *RSC Advances*.

[5] Zhao D. D., Wang J., Cui Y. J., Wu X. F. (2012). Pharmacological effects of Chinese herb aconite (Fuzi) on cardiovascular system. *Journal of Traditional Chinese Medicine*.

[6] Peng W.-W., Li W., Li J.-S. (2013). The effects of Rhizoma Zingiberis on pharmacokinetics of six *Aconitum* alkaloids in herb couple of Radix Aconiti Lateralis-Rhizoma Zingiberis. *Journal of Ethnopharmacology*.

[7] Ung C. Y., Li H., Cao Z. W., Li Y. X., Chen Y. Z. (2007). Are herb-pairs of traditional Chinese medicine distinguishable from others? Pattern analysis and artificial intelligence classification study of traditionally defined herbal properties. *Journal of Ethnopharmacology*.

[8] Wang S. P., Hu Y. Y., Tan W. (2012). Compatibility art of traditional Chinese medicine: from the perspective of herb pairs. *Journal of Ethnopharmacology*.

[9] Tan Y., Ko J., Liu X. R. (2014). Serum metabolomics reveals betaine and phosphatidylcholine as potential biomarkers for the toxic responses of processed *Aconitum carmichaelii* Debx. *Molecular BioSystems*.

[10] Sun D.-Z., Li S.-D., Liu Y., Zhang Y., Mei R., Yang M.-H. (2013). Differences in the origin of philosophy between Chinese medicine and western medicine: exploration of the holistic advantages of Chinese medicine. *Chinese Journal of Integrative Medicine*.

[11] Liang X. J., Li H. Y., Li S. (2014). A novel network pharmacology approach to analyse traditional herbal formulae: the Liu-Wei-Di-Huang pill as a case study. *Molecular BioSystems*.

[12] Shyur L.-F., Yang N.-S. (2008). Metabolomics for phytomedicine research and drug development. *Current Opinion in Chemical Biology*.

[13] Zhang A.-H., Sun H., Han Y. (2013). Ultraperformance liquid chromatography-mass spectrometry based comprehensive metabolomics combined with pattern recognition and network analysis methods for characterization of metabolites and metabolic pathways from biological data sets. *Analytical Chemistry*.

[14] Widodo A., Yang B.-S. (2007). Support vector machine in machine condition monitoring and fault diagnosis. *Mechanical Systems and Signal Processing*.

[15] Valentini G. (2002). Gene expression data analysis of human lymphoma using support vector machines and output coding ensembles. *Artificial Intelligence in Medicine*.

[16] Li L., Sun B., Zhang Q. (2008). Metabonomic study on the toxicity of Hei-Shun-Pian, the processed lateral root of *Aconitum carmichaelii* Debx. (Ranunculaceae). *Journal of Ethnopharmacology*.

[17] Song Y. Q., Zhang X., Dong Y. H., Dai L. P., Peng C., Xie X. F. (2015). Acute toxicity study on intragastric administration of different processed radix aconiti lateralis praeparata products to beagle dogs. *World Science and Technology/Modernization of Traditional Chinese Medicine and Materia Medica*.

[18] Sheela Sasikumar C., Shyamala Devi C. S. (2000). Protective effect of Abana ®, a poly-herbal formulation, on isoproterenol-induced myocardial infarction in rats. *Indian Journal of Pharmacology*.

[19] Khalid M. A., Ashraf M. (1993). Direct detection of endogenous hydroxyl radical production in cultured adult cardiomyocytes during anoxia and reoxygenation: is the hydroxyl radical really the most damaging radical species?. *Circulation Research*.

[20] Luna A., Villanueva E., Castellano M., Jimenez G. (1982). The determination of CK, LDH and its isoenzymes in pericardial fluid and its application to the post-mortem diagnosis of myocardial infarction. *Forensic Science International*.

[21] Avula P. R., Asdaq S. M., Asad M. (2014). Effect of aged garlic extract and s-allyl cysteine and their interaction with atenolol during isoproterenol induced myocardial toxicity in rats. *Indian Journal of Pharmacology*.

[22] Amani M., Jeddi S., Ahmadiasl N., Usefzade N., Zaman J. (2013). Effect of HEMADO on level of CK-MB and LDH enzymes after ischemia/reperfusion injury in isolated rat heart. *BioImpacts*.

[23] Babuin L., Jaffe A. S. (2005). Troponin: the biomarker of choice for the detection of cardiac injury. *Canadian Medical Association Journal*.

[24] Tonomura Y., Mori Y., Torii M., Uehara T. (2009). Evaluation of the usefulness of biomarkers for cardiac and skeletal myotoxicity in rats. *Toxicology*.

[25] Huang Z. F., Yi J. H., Chen Y., Liu Y. H. (2012). Influences of combination and ph of aconiti lateralis radix decoction on the six ester alkaloids contents. *Journal of Experimental Traditional Medical Formulae*.

[26] Ma Y. H., Li L., Ruan Y., Zhang H. Y., Ouyang J. P. (2011). Toxicity of heart, liver, kidney in the combination of Aconiti Lateralis Radix Praeparata and Trichosanthis Fructus. *Chinese Journal of Gerontology*.

[27] Bian B. L., Si N., Wang H. J., Yang J., He X. R. (2006). Study on the content changes of aconitine, hypaconitine and mesaconitine in the decoctions before and after Aconiti Lateralis Radix Praeparata combined with Fritillariae Thunbergii Bulbus. *Chinese Journal of Experimental Traditional Medical Formulae*.

